# Health and Oral Health-Related Quality of Life and the Associated Factors in Diabetic Patients

**DOI:** 10.7759/cureus.75269

**Published:** 2024-12-07

**Authors:** Mohammed Khalid Mahmood, Esra Mohammedameen, Salman Jaff, Handren A Kurda, Herve Tassery, Romain Lan, Delphine Tardivo, Balen H Qadir, Mohammed T Fatih, Mohammed A Abdulghfor

**Affiliations:** 1 Odontology, Aix-Marseille University, Marseille, FRA; 2 Internal Medicine, College of Medicine – University of Sulaimani, Sulaymaniyah, IRQ; 3 Pharmacology, College of Pharmacy, Komar University of Science and Technology, Sulaymaniyah, IRQ; 4 Orthodontics, College of Medicine – University of Sulaimani, Sulaymaniyah, IRQ; 5 Odontology, Timone Hospital, Aix-Marseille University, Marseille, FRA; 6 Prosthodontics, College of Dentistry, Komar University of Science and Technology, Sulaymaniyah, IRQ; 7 Periodontics, College of Dentistry, Komar University of Science and Technology, Sulaymaniyah, IRQ; 8 Oral and Maxillofacial Surgery, College of Medicine – University of Sulaimani, Sulaymaniyah, IRQ

**Keywords:** diabetes, health-related quality of life, oral health-related quality of life, quality of life, types 2 diabetes

## Abstract

Background: Diabetes mellitus, a chronic multi-systemic disease affecting various organs, may negatively influence health-related quality of life (HRQoL) and oral health-related quality of life (OHRQoL). This study aimed to investigate this association in a cross-sectional sample of Iraqi Kurdish diabetic patients.

Methods: Two hundred eighty-five type 2 diabetic patients participated in the survey. The EuroQol-5 Dimensions-5 Levels (EQ-5D-5L) and the Oral Health Impact Profile-5 (OHIP-5) instruments were used to measure HRQoL and OHRQoL, respectively.

Results: For the HRQoL, 75%, 64%, 46%, and 20% of the participants reported "some" problems in the fields of pain/discomfort, anxiety/depression, mobility, and interruption in usual activities, respectively. Concerning the OHRQoL, difficulty in chewing, finding less flavor in food, oral/dental pain, uneasiness about appearance, and difficulty in doing usual activities were the most complained fields of OHRQoL in 35%, 30%, 29%, 25%, and 20% of the patients respectively. Women had significantly worse HRQoL and OHRQoL compared to men. There was a positive, directly proportional, and significant correlation between HRQoL and OHRQoL (Pearson correlation test = 0.455, *p *= 0.000).

Conclusion: Sex, income, duration of diabetes, and smoking were the predictive modifying factors for both HRQoL and OHRQoL. From a public health point of view, raising health awareness is urgently needed among Iraqi diabetic patients for better diabetes management, a healthier lifestyle, and regular oral hygiene measurements.

## Introduction

Diabetes mellitus (DM) is a long-term metabolic condition that is caused by high blood glucose levels, insulin malfunction, and insulin deficiency. Chronic hyperglycemia leads to abnormalities and malfunctioning in the kidneys, neurological system, eyes, limbs, and cardiovascular system, among other body organs. According to estimates from the International Diabetes Federation, 215 countries had 537 million adults with diabetes in 2017. Additionally, the federation predicted that 783.2 million people globally will suffer from diabetes by 2045 [1]. Millions of individuals worldwide suffer from diabetes, which is a significant source of illness and death and a substantial economic burden on public health funding. Type 2 diabetes is influenced by a combination of environmental, genetic, and metabolic risk factors. These factors are interconnected. The most vulnerable people are those with a high risk of diabetes mellitus due to age, obesity, physical inactivity, and a strong family history [2].

One of the target areas for the negative effects of DM is the oral cavity and its numerous tissues. The most frequent oral symptoms of DM include a higher incidence of dental caries, a higher frequency of gingivitis and periodontitis and their destruction, a lower salivary flow, a higher risk of oral candidiasis, certain vesiculobullous lesions, a potential delay in wound healing, and a higher frequency of post-surgical infections [3].

A population's or an individual's current state of mind on both the positive and negative aspects of their life at any given time is referred to as their quality of life (QoL). Health-related quality of life (HRQoL) is a multifaceted idea commonly used to study the relationship between an individual's quality of life and health [4]. Moreover, expectations and satisfaction with care, a person's sense of self about their oral health, and their subjective appraisal of their functional and emotional well-being are all included in the complex concept known as oral health-related quality of life, or OHRQoL. It has broad applications in survey and clinical research. A vital aspect of general health and wellness is OHRQoL. In fact, it is recognized by the World Health Organization as a crucial part of the Global Oral Health Program (2003) [5].

Numerous investigations have been conducted on the HRQoL of diabetes patients, and thorough reviews can be found in the literature [6,7]. Different functional and well-being domains each contribute differently to overall quality of life, suggesting the need for a multidimensional measuring strategy. Because of this, the bulk of studies on the quality of life of diabetics have used multidimensional assessments that take into account social, psychological, and physical functioning and well-being. The two main methods for assessing HRQoL are the use of generic and disease-specific instruments. Research on diabetes patients has shown that these methods work well together and yield different types of information, with the generic instruments potentially offering more data than their disease-specific counterparts [4].

Furthermore, a number of studies examined the negative effects that oral health issues may have on emotional state, social status, psychological comfort, and physical function [8]. Thus, it is indisputable that dental and oral health is related with, and they have potential effects on overall quality of life. Moreover, diabetes was taken into account in a few of the initial investigations as a potential factor affecting OHRQoL [9].

Little is known about the impact of diabetes on QoL in Iraqi diabetic patients. Therefore, this article aimed to investigate the factors associated with HRQoL and OHRQoL among type 2 diabetic patients in Kurdistan, Iraq.

## Materials and methods

Study design and participants

Patients were recruited from the outpatient clinics in the Diabetes and Endocrine Center in Sulaimani, Kurdistan, Iraq, from January through May 2024 in a cross-sectional study. Of the 317 patients with type 2 diabetes who met the study's eligibility requirements, 285 chose to participate. They were asked to sign a permission and consent form. Lack of time and lack of desire to engage were given as reasons for declining by the non-willing patients. The study received ethical approval from the Institutional Review Board of the University of Sulaimani (no: 041B/2024).

Inclusion and exclusion criteria

Participants in the study had to be diagnosed as type 2 diabetes for at least six months, having at least 18 years old and willing to take part in order to be considered for inclusion. Patients with type 1 diabetes were excluded from the survey.

Data collection

Using a specially created questionnaire, patients' information on sociodemographic factors such as age, gender, education, income, and smoking status was gathered. The study collected data on various clinical and medication variables from patients' hospital charts or directly from the patients themselves. These variables included the duration of diabetes, family history, number of diabetic medications, presence of other chronic diseases and co-morbidities, BMI, physical activity, following a specific diet adjusted to diabetes, carbohydrate consumption, tooth brushing, tooth mobility and/or extraction due to diabetes, knowledge on the link between diabetes and oral/dental health, oral dehydration/mucositis and halitosis.

Instruments

The EuroQol-5 Dimensions-5 Levels (EQ-5D-5L) [10] and the Oral Health Impact Profile-5 (OHIP-5) [11] instruments were used to measure the HRQoL and OHRQoL respectively. EQ-5D-5L measures the following five health dimensions: mobility, self-care, usual activities, pain/discomfort, and anxiety/depression. Responses for each section include five levels: no problem, slight problem, moderate problem, severe problem, and unable to do/very severe problem. These answers were given a score from 0 to 4. The scores were summed up to give a total HRQoL score. The higher the score, the worse the HRQoL status. OHIP-5 measures the OHRQoL in five domains: difficulty chewing, oral/dental pain, feeling uncomfortable about the appearance of the dentition, feeling less flavor in food, and having difficulties doing usual jobs due to oral/dental problems. Responses for each of these sections include five categories: never, hardly ever, occasionally, fairly often, and very often. These answers were given a score from 0 to 4. The scores were summed up to give a total OHRQoL score. The higher the score, the worse the OHRQoL status. Both HRQoL and OHRQoL scores were treated as continuous variables.

Without any incentives, the patients independently filled out the surveys. Following a consultation with their physician, patients were interviewed in a private room at the outpatient clinic. Interviews lasted an average of fifteen minutes. The questionnaire was read aloud to the illiterate participants. The Kurdish translations of both questionnaires were used in the survey.

Data analysis

SPSS software (version 23; IBM SPSS, Armonk, NY) was used to code and analyze the data. The distribution of the HRQoL and OHRQoL indices among the sample under study and other potential confounders, including sociodemographic factors and disease-related traits, were all described using descriptive statistics. Multiple regression analysis was used to determine which variables best predicted the index scores in the study population using the EQ-5D-5L and OHIP-5 scores as dependent variables. The relative influence of each predictor on the outcome variable was expressed as beta values. P-values less than 0.05 were regarded as statistically significant.

## Results

An overall of 285 type 2 diabetic patients participated in the survey. The mean age of the participants was 52.5 ± 8.96 years, with a mean duration of diabetes of 5.9 ± 5.1 years. The majority of the patients (70.5%) had a positive family history of diabetes. Only 40% had another chronic disease. Most respondents took at least one diabetic medication (84.3%). The overwhelming majority of the participants were overweight or obese (96.8%). More than one-third of the sample (36.8%) experienced tooth mobility and/or extraction due to diabetic effects. Table 1 summarizes the sociodemographic, lifestyle, and diabetes-related variables described by the participants.

**Table 1:** Descriptive statistics of the participants (n = 285) BMI: body mass index; SD: standard deviation

Variables		n (%)	Mean ± SD
Sex	Male	105 (36.8)	
	Female	180 (63.2)	
Age (years)			52.5 ± 8.96
Education	Literate	165 (57.9)	
	Illiterate	120 (42.1)	
Income	Low	48 (16.8)	
	Medium	171 (60)	
	High	66 (23.2)	
Duration of diabetes (years)			5.9 ± 5.1
Family history	Yes	201 (70.5)	
	No	84 (29.5)	
Number of diabetic medications	No mediations	39 (13.7)	
	One	117 (41.1)	
	Two and more	123 (43.2)	
Smoking	Yes	36 (12.6)	
	No	249 (87.4)	
Other chronic diseases	Yes	153 (53.7)	
	No	132 (46.3)	
Physical activity	Yes	114 (40)	
	No	171 (60)	
Following a special diet adjusted for diabetes	Yes	177 (62.1)	
	No	108 (37.9)	
BMI	Normal	9 (3.2)	
	Overweight	102 (35.8)	
	Obese	171 (60)	
Tooth brushing	No	36 (12.6)	
	Sometimes	96 (33.7)	
	Daily	153 (53.7)	
Carbohydrate consumption	Low	192 (67.4)	
	Medium	66 (23.2)	
	High	27 (9.5)	
Tooth mobility and/or extraction due to diabetes	Yes	105 (36.8)	
	No	180 (63.2)	
Does diabetes affect dental and oral health?	Yes	129 (45.3)	
	No	156 (54.7)	
Halitosis	Yes	84 (29.5)	
	No	201 (70.5)	
Oral dehydration/mucositis	Yes	168 (58.9)	
	No	117 (41.1)	

Table 2 shows the total EQ-5D-5L score and the score for each level. Pain/discomfort was the most complained level, as 75.8% of the participants had "some" problems in this field. The second most affected field was anxiety/depression, with 64.2% of the patients reporting "some" problems. This was followed by mobility-related problems, with a mean of 0.91 ± 1.16. Usual activities were interrupted in almost half of the sample. Finally, 92.6% of the interviewed persons had no problems concerning their self-care.

**Table 2:** Distribution of health-related quality of life dimensions measured by EQ-5D-5L (n = 285) SD: standard deviation; EQ-5D-5L: EuroQol-5 Dimensions-5 Levels

Parameters	No problem (n (%))	Slight problem (n (%))	Moderate problem (n (%))	Severe problem (n (%))	Extreme problem (n (%))	Total score
						Mean ± SD
Mobility	153 (53.7)	51 (17.9)	45 (15.8)	27 (9.5)	9 (3.2)	0.91 ± 1.16
Self-care	264 (92.6)	15 (5.3)	6 (2.1)	0 (0)	0 (0)	0.09 ± 0.35
Usual activities	147 (51.6)	30 (10.5)	9 (3.2)	6 (2.1)	3 (1.1)	0.40 ± 0.84
Pain/discomfort	69 (24.2)	51 (17.9)	90 (31.6)	66 (23.2)	9 (3.2)	1.63 ± 1.17
Anxiety/depression	102 (35.8)	54 (18.9)	60 (21.1)	45 (15.8)	24 (8.4)	1.42 ± 1.33

Table 3 shows each domain's total OHIP-5 score and a separate score. Patients complained most about difficulty in chewing (35.8%). This was followed by the problem of "less flavor in food" with a mean of 0.72 ± 1.23. Out of 285 patients, 87 persons reported some dental/oral pain. Moreover, 24.2% of the participants reported concerns about their dentition's aesthetic appearance. Finally, only 20% of the patients said that oral and dental health-related issues caused some kind of interruption in performing their usual jobs. 

**Table 3:** Distribution of oral health-related quality of life dimensions measured by OHIP-5 (n= 285) SD: standard deviation; OHIP-5: Oral Health Impact Profile-5

Parameters	Never (n (%))	Hardly ever (n (%))	Occasionally (n (%))	Fairly often (n (%))	Very often (n (%))	Total score
						Mean ± SD
Difficulty in chewing	183 (64.2)	18 (6.3)	48 (16.8)	27 (9.5)	9 (3.2)	0.81± 1.20
Pain	198 (69.5)	27 (9.5)	45 (15.8)	15 (5.3)	0 (0)	0.57 ± 0.93
Uncomfortable about appearance	216 (75.8)	18 (6.3)	21 (7.4)	24 (8.4)	6 (2.1)	0.55 ± 1.07
Less flavor in food	201 (70.5)	21 (7.4)	15 (5.3)	39 (13.7)	9 (3.2)	0.72 ± 1.23
Difficulty in doing usual activities	228 (80)	18 (6.3)	21 (7.4)	15 (5.3)	3 (1.1)	0.41 ± 0.91

Concerning the sex differences, HRQoL total scores for women and men were 5.58 ± 3.11 and 2.46 ± 2.59, respectively. Moreover, OHRQoL total scores for women and men were 3.65 ± 4.20 and 2.08 ± 3.54, respectively. In both cases, the difference was statistically significant (Table 4).

**Table 4:** Comparison of HRQoL and OHRQoL scores between males and females HRQoL: Health-related quality of life, OHRQoL: Oral health-related quality of life

	n (%)	HRQoL	OHRQoL
		Mean ± SD	Mean ± SD
Male	105 (36.8)	2.46 ± 2.59	2.08 ± 3.54
Female	180 (63.2)	5.58 ± 3.11	3.65 ± 4.20
Total	285 (100)	4.43 ± 3.29	3.07 ± 4.0
p-value		0.000	0.002

In the multiple logistic regression model for EQ-5D-5L score, among the sociodemographic parameters, sex, income, smoking status, and physical activity independently and significantly affected the HRQoL outcome. Amid the diabetes-related factors, the duration of diabetes, following a special diet adjusted to diabetes, and carbohydrate consumption independently and significantly predicted the HRQoL outcome. No significant association was found between age, education, family history, number of diabetic medications, other chronic diseases, and BMI (Table 5).

**Table 5:** Multiple logistic regression analysis of factors predicting HRQoL HRQoL: Health-related quality of life

Parameters	Beta	t	p-value
Age	0.081	1.31	0.18
Sex	-0.5	-8.1	0.000
Education	-0.04	-0.8	0.42
Income	0.23	4.13	0.000
Duration of diabetes	0.14	2.53	0.012
Family history	0.06	1.16	0.24
Number of diabetic medications	-0.09	-1.59	0.11
Smoking	0.13	2.24	0.02
Other chronic diseases	0.05	0.87	0.38
Physical activity	-0.13	-2.33	0.02
Following an adjusted diet due to diabetes	0.16	2.86	0.005
BMI	-0.02	-0.46	0.64
Carbohydrate consumption	0.21	3.72	0.000

The multiple logistic regression analysis for the factors that affected the OHIP-5 score and eleven overall parameters independently and significantly predicted the OHRQoL score. Six of them were related to sociodemographic, non-diabetic, and lifestyle characteristics, namely age, sex, income, smoking, other chronic diseases, and tooth brushing. The other five parameters were related to diabetes, including duration of diabetes, number of diabetic medications, knowledge of the link between diabetes and oral/dental health, halitosis, and oral dehydration/mucositis. No significant association was found for education, family history, following a special diet adjusted for diabetes, carbohydrate consumption, tooth mobility and/or extraction due to diabetes (Table 6). Finally, we performed a correlation test between HRQoL and OHRQoL; the result was positive, directly proportional, and significant (Pearson correlation test = 0.455, p = 0.000).

**Table 6:** Multiple logistic regression analysis of factors predicting OHRQoL OHRQoL: Oral health-related quality of life

Parameters	Beta	t	p-value
Age	0.23	3.62	0.000
Sex	-0.28	-4.28	0.000
Education	0.04	0.77	0.43
Income	0.20	3.58	0.000
Duration of diabetes	-0.11	-1.80	0.048
Family history	-0.01	-0.18	0.85
Number of diabetic medications	-0.11	-1.99	0.04
Smoking	0.233	3.72	0.000
Other chronic diseases	0.15	2.59	0.01
Following a special diet due to diabetes	0.01	0.17	0.86
Tooth brushing	-0.11	-1.92	0.048
Carbohydrate consumption	0.07	1.29	0.19
Tooth mobility and/or extraction due to diabetes	0.05	0.85	0.39
Knowledge on the link between diabetes and oral/dental health	0.33	5.3	0.000
Halitosis	0.13	2.3	0.02
Oral dehydration/mucositis	0.11	1.93	0.049

## Discussion

Type 2 diabetes is a chronic multi-systemic disease affecting various organs. It is both biologically and epidemiologically plausible that it could negatively impact the QoL of patients through direct and indirect effects. The main systemic complications of diabetes include retinopathy, neuropathy, nephropathy, diabetic foot, and a delay in wound healing. In addition, more than 90% of diabetic patients were found to have oral manifestations. The most common oral manifestations of DM include an increase in the prevalence of gingivitis, periodontitis, and their destruction, increased chance of dental caries, reduced salivary flow, halitosis, increased odds for oral candidiasis, burning mouth syndrome, and some vesiculobullous lesions. Besides this kind of direct manifestations, also through the side effects of medications used to treat and control blood sugar levels in the normal range, DM can worsen the quality of life in patients [12].

In this research, we examined the effect of diabetes and the associated factors on QoL in an Iraqi sample. For the HRQoL, pain/discomfort was the most complained level as 75.8% of the participants had "some" problems in this field. This can be explained by the multi-systemic involvement of diabetes, which increases the sources of pain sensation. Our result is comparable with the 77% reported in a study from Bangladesh [13]. Pain was followed by problems in anxiety/depression since 24% of the participants showed "severe" to "extreme" problems related to anxiety/depression. The prevalence of depression and anxiety disorders is common among people with DM. However, this result appears to be higher than the 14% of generalized anxiety disorders reported in a systematic review pooling the global data [14]. Nearly half of the population stated that they had "some" problem with mobility. The debilitating impact of diabetes on the limbs and joints, especially in the elderly population, is a known fact [15]. Apart from these direct effects of diabetes on the limbs and joints, the overwhelming majority (~97%) of the studied population were overweight or obese, and only 60% reported being physically active.

Regarding the OHRQoL, the most complained domain by the patients was difficulty in chewing. Diabetic patients have a higher risk of periodontitis and dental caries [16]. This will, in turn, cause loss of teeth and difficulty in chewing. Indeed, ~37% of the sample reported mobility and/or extraction of at least one tooth due to diabetic effects. The second highest OHRQoL domain was finding less flavor in food. This can be explained by reduced salivation, which is frequently seen in diabetic patients. Besides, taste sensation may be reduced in diabetic patients even with normal sugar levels [17]. In addition, type 2 diabetes is typically seen over the age of 45 years, and a decrease in the sensation of taste beyond this age is common [18]. Oral/dental pain was the third complaint in the OHRQoL domain. Again, this can be related to increased odds of periodontitis, dental caries, and burning mouth syndrome in diabetic patients [19]. Finally, ~25% stated that they had "some" uneasiness with the appearance of their dentition. The high prevalence of teeth loss due to diabetic effects may have impacted this psychological malaise.

In the HRQoL results, half of the sample and in the OHRQoL findings, one patient of of five (20%) reported "some" interruption in performing usual activities. These interruptions will be reflected in the productivity of the individuals and will cause an economic burden for working diabetic patients. Employees with type 2 diabetes missed 4.2 more days of work than workers without the disease. Employees with DM had productivity costs that were 13.3% (680$) higher [20]. Moreover, oral problems increase in diabetic patients. Oral and dental problems, consequently, account for sickness-related absences, reduction of productive capacity in the workplace, and a higher economic burden on diabetic patients [12].

The sociodemographic and lifestyle-related factors, namely female sex, lower income, smoking, and physical inactivity, were independently and significantly predicting a worse HRQoL outcome. There is consistent evidence in the literature about the negative effects of lower income, smoking, and physical inactivity on the HRQoL in the general population as well as diabetic patients [4]. Among the diabetes-related factors, shorter duration of diabetes, following a special diet adjusted to diabetes, and lower carbohydrate consumption independently and significantly predicted a better HRQoL outcome. A longer duration of diabetes is associated with higher risks of diabetes-related complications and the prevalence of other chronic diseases [21], leading to a worse HRQoL level. Concerning the positive effects of following a special diet adjusted to diabetes and lower carbohydrate consumption, diet is known to be a crucial modifying factor in preventing and treating diabetes [22].

For the OHRQoL, eleven parameters significantly predicted the score overall. Six of them were related to sociodemographic, non-diabetic, and lifestyle characteristics. Higher age, female sex, lower income, smoking, presence of other chronic diseases, and no or lesser tooth brushing frequency were all related to a worse OHRQoL status. Other researchers have reported similar findings [9,23]. Especially, income was a significant parameter for both HRQoL and OHRQoL. A strong association between income and QoL has been documented both in high and low-income countries [24]. The other five parameters were related to diabetes. The shorter duration of the diabetes, lower number of diabetic medications, not having knowledge of the link between diabetes and oral/dental health, not having halitosis, and not suffering from oral dehydration/mucositis were all statistically associated with a better OHRQoL score. The special impact of diabetes-related factors on the OHRQoL status has been emphasized in some other research [25]. Our study shows that patients who know and affirm a relation between diabetes and oral health have gained this knowledge from a bad experience with oral health; therefore, they have a worse state of OHRQoL.

In the present study, regular tooth brushing was significantly associated with a better OHRQoL; confirming again an old and well-known fact [26]. Also, among the studied population, self-perceived mouth odor and suffering from oral dehydration/mucositis were significantly associated with a lower OHRQoL. In the previous research, a relation between self-reported halitosis and HbA1c level among type 2 DM patients has been documented [27]. The correlation between halitosis and elevated blood sugar levels could be explained by a number of plausible factors, including the phenomenon of ketoacidosis linked to poorly controlled diabetes, the common manifestation of dry mouth among diabetics, the increased risk of infections among diabetics, and an increase in the number of anaerobic microbiota on the tongue that releases products containing sulfur. As stated earlier, ~37% of the participants reported tooth mobility and/or extraction due to diabetes. This emphasizes the need for an earlier diagnosis of periodontitis. Indeed, several papers have investigated the positive effect of oral hygiene measures and periodontal treatment on improving periodontal health [28].

Despite the previous aforementioned points, a recent systematic review and meta-analysis on the effect of diabetes on OHRQoL stated that while it appears that DM can result in functional limits, physical pain, psychological discomfort, and problems related to diabetes negatively impact well-being, no statistically significant correlation was found between diabetes mellitus and OHRQoL [29].

In both HRQoL and OHRQoL indices, women scored higher, indicating a statistically significant worse condition compared to men. Findings from other Asian countries were also in agreement with the current result [2,4]. From the perspective of Iraq, men's greater levels of physical activity and comparatively better social lives may help explain their high degree of contentment with their quality of life. In addition, women are more inclined to express themselves and lament their low quality of life. According to earlier research, men reported a better quality of life, were more confident about their ability to control their blood sugar, and were less likely, compared to women, to experience anxiety [30].

Interestingly, in the multivariable analysis for HRQoL, no significant association was found for age, education, family history, number of diabetic medications, other chronic diseases, and BMI. Moreover, in the multiple regression analysis for OHRQoL, no significant association was found for education, family history, following a special diet adjusted for diabetes, carbohydrate consumption, tooth mobility, and/or extraction due to diabetes. Both biologically and epidemiologically, all of these parameters may have significant effects on the HRQoL and OHRQoL in diabetic populations or certain individuals; however, due to the sampling technique and the preferences of the studied population, they were only revealed to be statistically less significant than the others.

There was a positive, directly proportional, and significant correlation between HRQoL and OHRQoL (Pearson correlation test = 0.455, p = 0.000). This emphasizes the mutual interdependency between systemic and oral health. Significant associations between oral health and a number of systemic diseases have been established in the literature [8]; hence, it is natural to observe a directly proportional correlation between HRQoL and OHRQoL.

The study's strengths were its population size, social diversity, and the broad range of diabetes-related and socioeconomic characteristics examined. Furthermore, we assessed both OHRQoL and HRQoL in a group of individuals with diabetes. One study limitation was the use of self-reported health status, socioeconomic, and lifestyle-related parameters.

## Conclusions

Among Iraqi Kurdish diabetes patients, pain/discomfort, anxiety/depression, and mobility were the most affected fields of HRQoL, while difficulty in chewing, finding less flavor in food, and oral/dental pain were the most complained fields of OHRQoL. Women had significantly worse HRQoL and OHRQoL compared to men. There was a positive, directly proportional, and significant correlation between HRQoL and OHRQoL. Sex, income, duration of diabetes, and smoking were the predictive modifying factors for both HRQoL and OHRQoL. However, further case-control and longitudinal studies are required to explore the exact nature and extent of the association. From a public health point of view, raising health awareness is urgently needed among Iraqi diabetic patients for better management of diabetes, a healthier lifestyle, and regular oral hygiene measurements.

## Appendices

**Table 7:** EQ-5D-5L instrument Under each heading, please tick the ONE box that best describes your health TODAY. EQ-5D-5L: EuroQol-5 Dimensions-5 Levels

MOBILITY	
I have no problems in walking about	⚠
I have slight problems in walking about	⚠
I have moderate problems in walking about	⚠
I have severe problems in walking about	⚠
I am unable to walk about	⚠
SELF-CARE	
I have no problems washing or dressing myself	⚠
I have slight problems washing or dressing myself	⚠
I have moderate problems washing or dressing myself	⚠
I have severe problems washing or dressing myself	⚠
I am unable to wash or dress myself	⚠
USUAL ACTIVITIES (e.g. work, study, housework, family or leisure activities)	
I have no problems doing my usual activities	⚠
I have slight problems doing my usual activities	⚠
I have moderate problems doing my usual activities	⚠
I have severe problems doing my usual activities	⚠
I am unable to do my usual activities	⚠
PAIN / DISCOMFORT	
I have no pain or discomfort	⚠
I have slight pain or discomfort	⚠
I have moderate pain or discomfort	⚠
I have severe pain or discomfort	⚠
I have extreme pain or discomfort	⚠
ANXIETY / DEPRESSION	
I am not anxious or depressed	⚠
I am slightly anxious or depressed	⚠
I am moderately anxious or depressed	⚠
I am severely anxious or depressed	⚠
I am extremely anxious or depressed	⚠

**Table 8:** OHIP-5 instrument OHIP-5: Oral Health Impact Profile-5

Never	⚠
Hardly ever	⚠
Occasionally	⚠
Fairly often	⚠
Very often	⚠

Never	⚠
Hardly ever	⚠
Occasionally	⚠
Fairly often	⚠
Very often	⚠

Never	⚠
Hardly ever	⚠
Occasionally	⚠
Fairly often	⚠
Very often	⚠

Never	⚠
Hardly ever	⚠
Occasionally	⚠
Fairly often	⚠
Very often	⚠

Never	⚠
Hardly ever	⚠
Occasionally	⚠
Fairly often	⚠
Very often	⚠
